# Bridging the scales in high-throughput dielectrophoretic (bio-)particle separation in porous media

**DOI:** 10.1038/s41598-018-28735-w

**Published:** 2018-07-11

**Authors:** Georg R. Pesch, Malte Lorenz, Shaurya Sachdev, Samir Salameh, Fei Du, Michael Baune, Pouyan E. Boukany, Jorg Thöming

**Affiliations:** 1 University of Bremen and Center for Environmental Research and Sustainable Technology, Chemical Engineering: Recovery and Recycling (VdW), Bremen, Germany; 2 Delft University of Technology, Department of Chemical Engineering, Delft, The Netherlands

## Abstract

Dielectrophoresis (DEP) is a versatile technique for the solution of difficult (bio-)particle separation tasks based on size and material. Particle motion by DEP requires a highly inhomogeneous electric field. Thus, the throughput of classical DEP devices is limited by restrictions on the channel size to achieve large enough gradients. Here, we investigate dielectrophoretic filtration, in which channel size and separation performance are decoupled because particles are trapped at induced field maxima in a porous separation matrix. By simulating microfluidic model porous media, we derive design rules for DEP filters and verify them using model particles (polystyrene) and biological cells (S. cerevisiae, yeast). Further, we bridge the throughput gap by separating yeast in an alumina sponge and show that the design rules are equally applicable in real porous media at high throughput. While maintaining almost 100% efficiency, we process up to 9 mL min^−1^, several orders of magnitude more than most state-of-the-art DEP applications. Our microfluidic approach provides new insight into trapping dynamics in porous media, which even can be applied in real sponges. These results pave the way toward high-throughput retention, which is capable of solving existing problems such as cell separation in liquid biopsy or precious metal recovery.

## Introduction

Separation of micron and sub-micron particles from liquid or according to their properties is a highly relevant topic in a variety of fields, for example, in pharmaceutical production^1,2^, in diagnostics and analytics^3^, or in the recovery of materials^4,5^. Classical examples for pressing separation problems are the detection of circulating tumor cells (CTC) from whole blood^6^ or the recovery of precious metals from electronic scrap in urban mining^5^. While there is a potpourri of microfluidic techniques for the detection of circulating tumor cells^6^, most of them (that are based on either inertia or size exclusion) require size differences between the blood cells and the circulating tumor cells. Depending on the type of cancer, the assumption that CTCs are sufficiently larger than blood cells is questionable^3,6^. Our second example is the recovery of precious metals from a mixture of metal, metal oxides, and polymers. Here, the first step is usually shredding of the mixture which generates a lot of dust. The large solid particles are subsequently separated using standard methods whereas the dust fraction is lost, albeit it contains particles in the μm size range composed of a variety of highly valuable materials^7^. Further, most of the established techniques for the separation of the larger particles are either costly or environmentally unfriendly^8^. Dielectrophoresis (DEP)^9,10^, a highly selective particle manipulation technique, is suited to solve such difficult separation tasks.

Up to now, DEP has mostly been used in microfluidic devices to solve biomedical separation problems^11^ in the μL min^−1^-scale. As an example, DEP has proven to be very effective in terms of selectivity for the separation of biological cells such as CTCs from blood^12^. However, currently, the throughput of such devices is below the threshold of what is considered acceptable for the analysis of clinical samples^3^. A low throughput combined with a high selectivity is one of the general characteristics of contemporary DEP devices. Hence, to treat samples of preparative or industrial quantities, it is necessary to increase the throughput of DEP devices significantly.

Dielectrophoresis is based on the action of an inhomogeneous electric field on a temporary induced dipole (or multipole^13,14^). The dielectrophoretic force in an ac electric field depends on the volume of the particle, on its relative polarizability, and on the spatial change of the electric field. In the point-dipole approximation this force is expressed as

1 $$\langle {\overrightarrow{F}}_{{\rm{DEP}}}\rangle =\frac{1}{4}\pi {d}_{{\rm{P}}}^{3}{\rm{Re}}[K]\nabla {|{\overrightarrow{E}}_{{\rm{RMS}}}|}^{2}.$$

with the time-averaged DEP force $\langle {\overrightarrow{F}}_{DEP}\rangle$, the del operator ▽ = (∂/∂*x*, ∂/∂*y*, ∂/∂*z*), which gives the gradient of a scalar field, the particle diameter *d*_P_ , the real part of the complex Clausius-Mossotti function Re[*K*], and the root-mean-square electric field vector ${\overrightarrow{E}}_{RMS}$. The real part of the Clausius-Mossotti function gives the relative polarizability of the particle in the medium. It is dependent on the permittivities and conductivities of the medium and the particle as well as on the frequency of the applied ac field. It is bound between −0.5 and 1. If the particle is less polarizable than the surrounding medium, it moves towards regions of low field (against the gradient), which is termed negative DEP (nDEP). Conversely, if the particle is better polarizable than the medium it moves in direction of the gradient towards regions of highest field strength, termed positive DEP (pDEP). The dependence of Re[*K*] on the particle properties explains the versatility of DEP: It is possible to solve difficult separation tasks since particles of equal size and density could be moved into different directions in the electric field due to different polarizabilities.

Particle movement due to dielectrophoresis can only occur when the field has a gradient (Eq. (1)) and, in order to ensure forces large enough for trapping or separation, with decreasing particle size this gradient needs to increase. One way for generating the field gradients is to use micro-electrode structures located at some position in a microchannel (electrode-based dielectrophoresis). This is done, for instance, in the device for the separation of circulating tumor cells from whole blood presented in ref.^15^ or in a recent report on the separation of onion-like carbon^16^. The micro electrodes employed in such devices are usually in the same size range as the particles to be separated. Depending on the operation mode, sorting or trapping, particles that experience positive or negative DEP will be directed toward different outlets due to the presence of the field gradient or particles that experience positive DEP will be immobilized in points of maximum electric field that serve as particle traps.

As a consequence a high density of particles that experience positive DEP might cause electrode fouling after some time of continuous operation^17,18^. Such devices usually present good trapping capabilities at comparably high throughputs. Their scale-up possibility—that is, increasing the throughput by increasing the channel size—is limited due to rapid decrease of $\nabla {|\overrightarrow{E}|}^{2}$ with increasing distance from the electrode array. This limits the overall possible channel size. A different approach to generate the necessary field gradients is insulator-based or electrodeless DEP (iDEP)^17,18^. Here, the electric field is generated by two electrodes that are located outside of the main separation channel. The originally homogeneous field is disturbed at field obstacles in the channel. A very common arrangement is an array of insulating posts. Such devices have been used, for example, for the separation of live and dead bacteria^19^, for the trapping of DNA^20^, or the isolation of proteins^21^. In the traditional operation mode^22–24^, particles are driven through the post array by electrokinetic movement (that is, electrophoresis and electro-osmosis). A negative DEP force acts against this electrokinetic movement. At each line of posts, there exists a point where the DEP force matches the electrokinetic force and particles become immobilized (that is, electrokinetics drives them in one direction and DEP drives them in the opposite direction). Depending on the particle type, this immobilization requires a different threshold voltage. This enables effective separation between different types of particles by a careful tuning of the applied voltage. As nicely argued by Pethig in his 2017 book (ref.^10^, Sec. 10.4.2.1), iDEP devices are much cheaper to produce than classical electrode-based devices and they show a very high discrimination ability. Their throughput, however, is low compared to classical electrode-based designs and their voltage demands are much higher. Application for iDEP is in the point-of-care diagnostic or in cases where only low volumes of very pure product are required.

The vast majority of all contemporary dielectrophoretic applications focus on the particle manipulation in lab-on-a-chip devices at low or very moderate throughputs (below 1 mLh^−1^). Some attempts have been made in order to increase the throughput^25–27^, however, particle manipulation in the preparative scale remains an issue in both, electrode-based and insulator-based DEP applications^28,29^. Somehow similar to iDEP is dielectrophoretic filtration. Here, due to the action of a pump, particle suspension flows through a macroscopic medium, which creates the electric field gradient, instead of microscopic pillar fields. Particles that experience positive DEP will then become trapped at points of maximum electric field inside of the filter medium. Since such filter media are random and macroscopic, they contain a literally infinite amount of traps.

Rather recent publications show that this concept is successful for the separation of yeast cells (about 5 μm) from water^30–32^ at moderate throughputs of 6 mLh^−1^. Earlier reports show that the concept of DEP filtration can be used for the separation of technical particles at very high throughputs of up to 180 Lh^−1^ or 16 m^3^m^−2^ h^−1^ ^33,34^ (albeit separating rather large particles with diameter >50 μm from non-conductive liquids at incredible voltage requirements of up to 12 kV). In our own proof-of-principle study, we were able to separate 340 nm layer-by-layer assembled nanocapsules using a monolithic polyethylene sponge as filter medium^2^ at throughputs of 60 mLh^−1^. All above-mentioned papers on DEP filtration never went past the proof-of-principle stage. Their efforts were more focused on the device operation than on scrutinizing the trapping dynamics in the filter and they especially never investigated the influence of the porous medium on the separation. Apart from our study^2^, all DEP filter reports used silica glass beads as filtration matrix, whereas we used a monolithic sponge. Interestingly, two recent publications from our group indicate that the trapping dynamics are directly influenced by the pore structure^35^ and pore size^36^ of the porous medium.

The main reason for the high throughput achieved in the filtration devices compared to conventional microfluidic devices usually used in DEP is the possibility to use a separation matrix with a large fluidic cross section. Electrode-based microfluidic devices can only have a maximum fluidic cross section in order to generate high enough field gradients^37^; very similar, classical polydimethylsiloxane-based microfluidic devices can only have a maximum channel height, limited by the production mechanism^38^. Instead, we propose that by employing a real porous medium as a filtration matrix we can emulate a vast number of parallel microchannels (thus the fluidic cross section of such devices is much larger) while simultaneously being easy to handle and cheap to produce.

**Figure 1:**
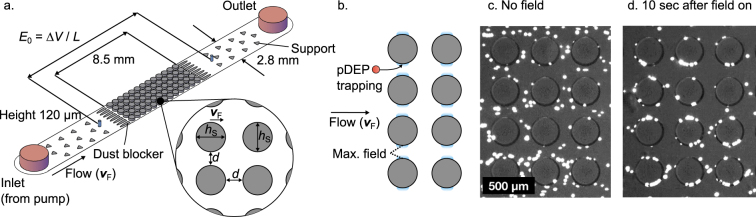
Scheme of the microfluidic system that serves as model porous medium (**a**). The microchannels have a height of 120 μm and a width of 2.8 mm. An array of insulating posts with cross-sectional diameter *h*_S_ is located in the center of the channel. They are placed a distance of *d* apart. The entire array has a length of 8 mm. The fluid velocity vector ${\overrightarrow{v}}_{{\rm{F}}}$ results from the pressure-driven volume flow *Q* through the channel. Elongated dust blockers are located in front and behind of the post array. The electric field *E*_0_ = Δ*V*/*L* across the array is generated by applying a voltage Δ*V* using two platinum electrodes (blue). Fluid inlet and outlet is realized using PTFE tubing (red) and fluid transport is achieved using a syringe pump. Triangular support structures are located in the channel to prevent it from collapsing. Principle of positive DEP (pDEP) particle trapping in the post array (**b**). The electric field is disturbed by the posts so that it shows maxima at the openings in each line of posts at the points that are on a line perpendicular to the flow and applied field direction. Particles experiencing positive DEP will become immobilized at these field maxima that act as particle traps. Snapshot of a microchannel with polystyrene model particles without (**c**) and ten seconds after application of the electric field (**d**); see also Supplementary movie S1.

As a consequence of our preliminary DEP filtration work^2^, we here scrutinize the filtration process by simulative and experimental investigation of particle trapping in model porous media. This allows for gaining a better understanding of the underlying phenomena and their quantification. By this we can relate separation performance with operational parameters and geometrical dimensions. A quasi two-dimensional array of insulating posts in a microfluidic channel is employed as porous medium (Fig. 1a) and particles are transported through the channel due to pressure-driven flow. This gives in principle the same trapping dynamics but the structure is much easier to describe. Using standard finite element (FE) simulations (trajectories of mass-less particles through a stationary fluid and electric field), we investigate the influence of key design (post spacing and post size) and operational (throughput, field strength, and particle diameter) parameters on the separation. We verify key results by experiments using polymeric model particles in microfluidic polydimethylsiloxane (PDMS) channels. This allows for direct visualization of the occurring processes. Further, by separating live yeast cells from salt buffer we show that the predictivity of our model is also valid for biological samples. To demonstrate the direct scale-up possibility of our model, we separate yeast cells from salt buffer using positive DEP in real alumina sponges at high throughputs (of up to 600 mLh^−1^). We demonstrate that the scaled-up process shows similar parameter dependencies as the separation process in the model filter. The direct scale-up possibility using real samples is an important step towards actual high-throughput DEP filtration that could tackle a wide range of current (cell-)separation challenges, such as the separation of circulating tumor cells from whole blood^3^.

## Results and Discussion

We will first describe results that were obtained using the microfluidic devices before we present results from the filtration setup. We start by describing the visible observation of the particle behavior in the microfluidic devices with and without electric field. Afterwards, we will discuss the experimentally determined and the simulated separation efficiencies. We will determine how reliable the predictions by simulations are for a variety of input parameters. Then, we will derive straight-forward design rules for microfluidic particle separators and show that they are applicable for model particles (polystyrene) and real biological samples (yeast). We will then transfer these design rules towards macroscopic separators that can operate at high throughput utilizing the same physical separation mechanism.

### Working principle

Under absence of an electric field polystyrene (model) particles moved through the channel relatively unaffected (Fig. 1c). That means, in rare instances particles became trapped on the PDMS surface; this was at no time observed but instead some immobilized particles could be found on surfaces after using a channel for some time.

When the electric field was turned on, particles started to accumulate on the surface of the post at the points perpendicular to the flow and field direction (Fig. 1d). The particle accumulation was obvious from the appearance of bright spots but it was not possible to tell of how many particles a single bright spot was composed. With increasing duration, more and more bright spots appeared on the posts. Particles preferentially accumulated slightly above or below the narrowest points in each line of posts. When the field was turned off, the particles readily desorbed from the surface and were re-entrained by the fluid. Depending on the throughput it could take up to a minute until all particles were re-suspended in the fluid flow. Especially at high flow rates, particles were not safely immobilized on the surface of the post but sometimes started to hop along from one post to the next. This effect was linked to a low separation efficiency.

The applied electric field is disturbed at each line of posts, generating an inhomogeneous field with maxima at the narrowest points of each post that are on a line perpendicular to the applied field (Fig. 1b, see also Methods section for a surface plot of the field distribution in the channel). Using a picturesque description, the field lines have to squeeze through the openings in each line of posts. Consequently, these field maxima serve as particle traps that attract the particles from the fluid stream. Depending on the balance between the DEP force due to the traps and the drag force due to the liquid, more or less particles are attracted and become immobilized on the posts’ surface. By counting the number of particles (using an in-house Matlab routine, see Methods section) that go into the channel and comparing this to the number of particles that exit the channel, it is possible to derive a separation efficiency (that is the percentage of particles that stays in the filter, see Methods section for an equation).

### Applied electric field strength, throughput, and post spacing

**Figure 2:**
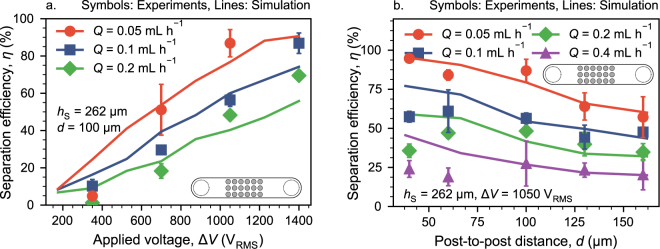
Separation efficiency in the microfluidic devices at different throughputs *Q* as a function of applied voltage Δ*V* at constant post spacing *d* = 100 μm (**a**) and (**b**) as a function of post-to-post spacing *d* at constant Δ*V* = 1050 V_RMS_. Post diameter is *h*_S_ = 262 μm in all cases. The particle size is *d*_P_ = 1 μm. Lines represent simulated separation efficiencies and points experimental data. The error bar represents one standard error. A relative polarizability of Re[*K*] = 0.52 was assumed for the simulation to match experiments and simulations. Due to large differences between the experiments and the simulation the line for *Q* = 0.4 mLh^−1^ has been omitted from panel a. The full figure can be found as Fig. S1a in the supplementary information. We have only experimentally verified post spacings up to 160 μm. Nevertheless, we also simulated larger spacings. Panel b with simulation results up to *d* = 500 μm can be found in the supplementary information (Fig. S1b). The Methods section gives a detailed explanation on how we obtained the values of Re[*K*]. Error bars show one standard error in all cases.

To understand the behavior of the separator and to assess the reliability of our simulations, we firstly simulated separation efficiencies as a function of throughput *Q*, applied voltage Δ*V* and post-to-post spacing *d*. The simulations numerically solved for particle trajectories under application of Eq. (1) in combination with numerical solutions of Stokes’ and Laplace’s equation (with COMSOL). We then verified the simulations by experimental comparison. For this, we used monodisperse 1 polystyrene (PS) particles and separated them from low-conductive KCl solution; this is our model system. At constant spacing *d* = 100 μm, the separation efficiency increased with Δ*V* and decreased with *Q* (Fig. 2a). For throughputs from *Q* = 0.05–0.2 mL h^−1^ the simulations matched the experimentally determined separation efficiencies quite well at moderate and high applied voltages (700–1400 V_RMS_). This is quite remarkable, because we applied no fit except for the real part of the Clausius-Mossotti factor, Re[*K*] (see Methods section on the procedure of how to obtain Re[*K*]). The found value for Re[*K*] falls well within the reasonable range. Other approaches to match experimental and simulated results in insulator-based DEP devices require a non-physical correction factor that could be as high as 1000 in some cases. In our case, however, the fitted Re[*K*] results in values of 0.52 (in case of polystyrene) and 0.18 (yeast) which are in good agreement with estimates from literature^39,40^. At low voltages (350 V_RMS_) and at high throughputs (*Q* = 0.4 mLh^−1^, only shown in supplementary information as Fig. S1a), the simulation tended to over-predict the experiments.

The separation efficiency as a function of post-to-post spacing *d* behaves similarly (Fig. 2b): At moderate and large spacings, *d* ≥ 100 μm, the simulations were able to predict the experiments with good accuracy. At narrow spacing, the simulation overpredicted the experiment. The deviation increased with increasing *Q*. In other words, the simulation predicted monotonically increasing *η* with decreasing *d* whereas the experiments suggested that there is an optimal spacing around *d* = 100 μm.

We assume that this deviation is due to the neglect of the particles’ finite size in the simulation. That is, the DEP force due to the particle trap is not strong enough to trap the particle irreversibly at the post at small *d* (a small *d* causes an unfavorable shift of the force balance at the post’s surface from DEP to drag), so it starts to hop along from post to post in the experiments. We have performed a series of further tests to support this assumption and the results can be found in the supplementary information (Sec. 2).

### Separator design rules

**Figure 3:**
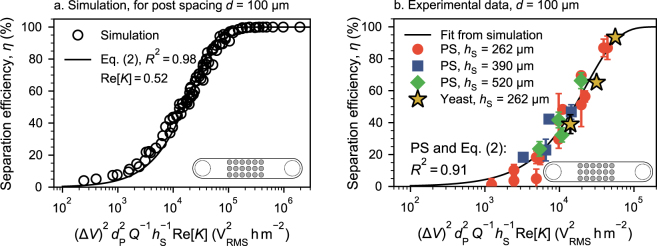
Separation efficiency *η* as a function of $\bar{x}={({\rm{\Delta }}V)}^{2}{d}_{{\rm{P}}}^{2}{h}_{{\rm{S}}}^{-1}\,{Q}^{-1}{\rm{Re}}[K]$. (**a**) Simulated separation efficiencies (empty symbols) for a variety of parameters *Q* = 0.05–0.1 mLh^−1^, *d*_P_ = 0.2–1 μm, Δ*V* = 350–4000 V_RMS_, and *h*_S_ = 130–1200 μm at Re[*K*] = 0.52 and *d* = 100 μm. The fit according to Eq. (2) shows a high *R*^2^ of 0.98 using Re[*K*] = 0.52. (**b**) Comparison of the fit from panel (a) with experimental data obtained using 1 μm polystyrene (PS) particles. Using the same $C=-24.6\times {10}^{3}{{\rm{V}}}_{{\rm{RMS}}}^{2}{{\rm{hm}}}^{-2}$ and comparison with the PS experiments gives a slightly lower *R*^2^ of 0.91. The red circles of panel b are equal to the data points presented in Fig. 2a. The blue and green data points at *h*_S_ = 390 μm and 520 μm were recorded at Δ*V* = 700–1050 V_RMS_ and *Q* = 0.1–0.2 mLh^−1^. Particle size *d*_P_ = 1 μm in all experiments. Again, a value of Re[*K*] = 0.52 was assumed for the PS particles to match the experiments with the simulation. The yellow stars show the separation efficiency of yeast cells (*d*_P_ = 5 μm, *Q* = 0.6 mLh^−1^, *h*_S_ = 262 μm and Δ*V* = 700–1050 V_RMS_). Here, a value of Re[*K*] = 0.18 was assumed to obtain a match between the fit and the experimental data. The Methods section gives a detailed explanation on how we obtained the values of Re[*K*] for yeast and PS. In all cases, error bars show one standard error. If error bars are not visible, they are smaller than the symbols. A version of panel b containing a numbering scheme and a corresponding table that contains the respective *h*_S_ and Δ*V* values can be found in the supplementary information (Fig. S12 and Table S2).

We know from a previous study (that investigates only fluid drag and DEP) that the trapping potential of a single post in the entire array has a quadratic dependence on the applied field strength and particle size to be separated and a linear reciprocal dependence on the volume flow and the post diameter^36^. The simulations performed here do not include any dispersion effects and only consider DEP and drag. Hence, when plotting the separation efficiency *η* for similar data as shown in Fig. 2 against ${({\rm{\Delta }}V)}^{2}{d}_{{\rm{P}}}^{2}{h}_{{\rm{S}}}^{-1}{Q}^{-1}{\rm{Re}}[K]$ all points should collapse on a single line (Fig. 3a). The linear dependence on Re[*K*] was added here to able to compare particles with different relative polarizabilities. This dependence is valid as long as the spacing *d* is constant. This was tested for a thoroughly mixed combination of all involved parameters (see figure legend). The separation efficiency can then be calculated as

2 $$\eta =100(1-\exp \frac{\overline{x}}{C})$$

with the parameters $\bar{x}={({\rm{\Delta }}V)}^{2}{d}_{{\rm{P}}}^{2}{h}_{{\rm{S}}}^{-1}{Q}^{-1}{\rm{Re}}[K]$ and the constant *C* that is a function of the post-to-post spacing *d*, $C=-\,2.46\times {10}^{8}{{\rm{V}}}_{{\rm{RMS}}}^{2}{{\rm{hm}}}^{-3}\cdot d$. Several post spacings were investigated (Fig. S5 in the supplementary information), shown here is *d* = 100 µm using $C=-\,24.6\times {10}^{3}{{\rm{V}}}_{{\rm{RMS}}}^{2}{{\rm{hm}}}^{-2}$ accordingly. We fitted Eq. (2) on the simulated data using the factor *C* as fitting factor and assuming Re[*K*] = 0.52 for PS particles (the function *C* = *f*(*d*) is obtained by fitting *C* for different *d* and then by applying another fit for the function *f* ).

In all cases *C* is negative. A value of *C* close to 0 indicates a geometry well-suited for particle separation (that is, with increasing magnitude of *C*, the S-shape of the curve where *η* switches from 0 to 100% moves towards larger values of $\bar{x}$). Hence, with increasing *d*, *C* becomes more negative, and the separation efficiency decreases (consistent with Fig. 2b).

To predict the separation efficiency for dielectrophoretic filter chips using an arbitrary parameter combination, we solely need Eq. (2). We consider this to be straight-forward design rules for dimensioning dielectrophoretic filter chips. This requires that the simulation results are sufficiently reliable as discussed in the supplementary information (Sec. 2). We will now show, that this relation also holds for experimental data using model particles and real biological samples (yeast cells). We further show that the same relations hold, to a certain extend, for real porous media (macroscopic alumina foams).

In principle, this fit is valid for all sorts of particles, as long as the dipole approximation is sufficiently valid (Eq. (1)). Different particle types can be accounted for by varying *d*_P_ and Re[*K*] in $\bar{x}$. A comparison of the fit from Fig. 3a with the experimentally determined separation efficiencies for 1 μm polystyrene model particles (at three different *h*_S_ = 262 μm, 390 μm, and 520 μm and different values of Δ*V* and *Q*) revealed a good accuracy of the prediction (Fig. 3b). Again, this is remarkable, because only Re[*K*] was required as a fitting parameter and the resulting value of Re[*K*] = 0.52 falls well within reasonable range (see Method section). The ${d}_{{\rm{P}}}^{2}$ dependence in $\eta (\bar{x})$ was not experimentally investigated because a change in *d*_P_ is always accompanied by a change in Re[*K*] due to the nature of the effective conductivity of polystyrene particles^39^. It is thus not possible to solely change *d*_P_ and we could easily fit all resulting data points to the existing simulation fit by assuming some (reasonable) value of Re[*K*]. Albeit being one of its key properties, investigating the non-selective size dependency of the separation efficiency at this stage does not yield new scientific insight. This is because size is not an independent variable and we cannot change it without inevitably changing Re[*K*] (which is our fitting factor).

We were further able to demonstrate that this fit can be readily transferred to other particle systems by separating yeast cells from KCl buffer solution (Fig. 3b, stars), which is a common example for DEP bio-particle separation^41–43^. These baker’s yeast cells have an approximate size of *d*_P_ = 5 μm. Literature reports of their Re[*K*]-value greatly varies over the entire possible spectrum (−0.5 to 1 depending on frequency and medium conductivity), which is common for biological systems. We have assumed Re[*K*] = 0.18 in order to match the fit and the experimental separation efficiencies, which is a very reasonable assumption (cf. for example ref.^10^, Fig. 11.7, or ref.^40^, Fig. 2A, we use a medium conductivity of 2.5 × 10^−4^ Sm^−1^ and 15 kHz frequency). The match between different particle systems and the simulations is remarkable and shows that the predictive ability of the fit is not limited to a specific *model* particle but also valid for *real* particle systems.

What we have presented so far was limited to throughputs below 1 mLh^−1^ and allowed us to identify and characterize the pDEP model. For bridging the discussed throughput gap we would now like to show that the results using model porous media are easily transferred toward much higher throughputs in macroscopic filtration media.

### Scale-up: Cell separation in a high-throughput porous medium

**Figure 4:**
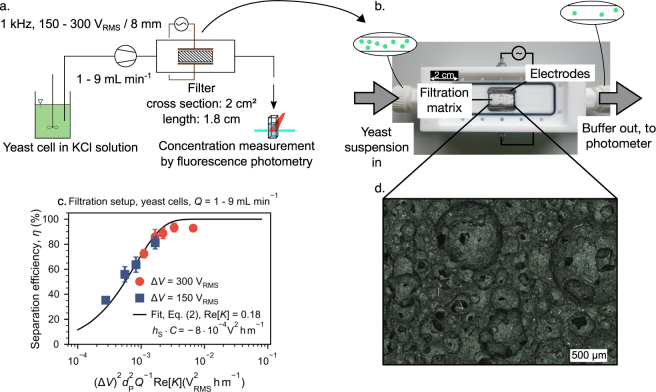
Separation of baker’s yeast cells using a macroscopic porous medium (**a** and **b**). The separation efficiency *η* plotted against ${\bar{x}}^{\ast }={({\rm{\Delta }}V)}^{2}{d}_{{\rm{P}}}^{2}{Q}^{-1}{\rm{Re}}[K]$ can be fitted by Eq. (2) using *C*⋅*h*_S_ as fitting parameters (**c**). Experiments were conducted using two different applied voltages Δ*V* = 150 V_RMS_ and 300 V_RMS_ and throughputs in the range of *Q* = 60–540 mLh^−1^. The employed mullite open cell foam had a fluidic cross-sectional area of 2 cm^2^, a volume based porosity of 83% and a *d*_50,3_ of 230 μm (panel d shows an optical microscopy image of a filter cross section).

In order to demonstrate that the design rules established for microfluidic channels are directly transferable to the scaled-up, more complex system (Fig. 4a), we performed yeast cell separation in ceramic filter (open cell foam, mullite, 83% porosity, median pore diameter of 130 μm, Fig. 4d, more details in the supplementary information, Sec. 5) that is sandwiched between two electrodes (Fig. 4b).

Again, we applied Eq. (2) for the separation efficiency *η* at high throughputs. Since the porous medium we use (Fig. 4c) was produced using a foaming technique, optical images of sectional views of the sponge do not allow for extraction of *h*_S_ (Fig. 4d). This is because–compared to the microfluidic channels that use circular posts as separation matrix (solid fraction)–the macroscopic filter has more resemblance with the inverse of a sphere packing (since the circular bubbles of the foam make up for the void fraction). As a consequence, we assume that the solid fraction is very thin in general (high porosity). In addition, in the macroscopic foam, the pore throat will have a sharp edge (as separation will occur in the pore windows) compared to circular pore throats found in the microchannels. Previous studies from our group showed, however, that the shape of the obstacle’s boundary itself has a negligible effect on the separation^36^. We assume that the shape (round vs. sharp) will have an influence on the fluid flow at higher Reynolds numbers. The comparably low volume flows applied here, however, yield only creeping flow through the pore structure (and thus the flow does not contain any vortices). According to the 1/*h*_S_ dependency of *η*, the very thin solid fraction found in the foam is beneficial. We have not yet identified a parameter analogous to *h*_S_ for the macroscopic foam. Consequently, we plotted all data against ${\bar{x}}^{\ast }={({\rm{\Delta }}V)}^{2}{d}_{{\rm{P}}}^{2}{Q}^{-1}{\rm{Re}}[K]$ and fitted Eq. (2) for *C*⋅*h*_S_ instead of *C*. As advertised, all points fall very nicely on the fit as already demonstrated for the separation in microfluidic channels. We believe that we could equally introduce a term describing the size of the filtration matrix into the abscissa parameter to make the results more general. This is a direct indication that the particle retention dynamics are equal in both systems and that the directly observable particle behavior in the transparent chips is equal to the non-observable behavior in the opaque filter!

The fit in Fig. 4c revealed a value of ${h}_{{\rm{S}}}\cdot {{\rm{C}}}_{{\rm{r}}{\rm{e}}{\rm{a}}{\rm{l}}}=-\,8\times {10}^{-4}\,{{\rm{V}}}_{{\rm{R}}{\rm{M}}{\rm{S}}}^{2}{{\rm{h}}{\rm{m}}}^{-1}$. We can compare this with the *C*_model_ from Fig. 3. Multiplying *C*_model_ with *h*_S_ = 262 μm yields ${h}_{{\rm{S}}}\cdot {{\rm{C}}}_{{\rm{m}}{\rm{o}}{\rm{d}}{\rm{e}}{\rm{l}}}=-\,6.44{{\rm{V}}}_{{\rm{R}}{\rm{M}}{\rm{S}}}^{2}{{\rm{h}}{\rm{m}}}^{-1}$, which is approximately four orders of magnitude more negative than the value of *h*_S_⋅*C*_real_. Again, a value of *C* (or *C*⋅*h*_S_ for that matter) close to 0 indicates a geometry more effective for particle separation (when C is close to 0, high values of *η* require smaller values of $\bar{x}$). Obviously, the main reason for this difference is the much larger cross section of the real filter medium (which is not separately considered in $\bar{x}$ but is incorporated in *C*). Comparing the cross section of the microchannel with the filter cross section reveals that an approximate amount of 750 microchannels would be required to achieve an equal cross section to the filter. Furthermore, some difference could be attributed to the different electrode alignment in the filter setup (perpendicular to the flow direction) that allows to decouple channel length and applied field strength. We thus have two beneficial effects, a long filtration matrix (in flow direction) combined with a strong applied field (perpendicular to the flow). A similar effect is used in the promising concept of contact-less dielectrophoresis^26,44,45^.

We note in passing that the results of Fig. 4c were achieved at throughputs of up to 9 mLmin^−1^ or 540 mLh^−1^ while the voltage requirements of 300 V_RMS_ are very modest compared to the 1400 V_RMS_ required in the microchannels. Processing more liquid while simultaneously operating at lower voltage compared to the microchannels is possible due to the vast difference in *h*_S_⋅*C* which is due to the larger fluidic cross section of the filter. Due to the interplay of all parameters in $\bar{x}$, operating at even lower Δ*V* would be possible when the throughput *Q* would be decreased or vice versa. To our own surprise, using a back-of-an-envelope calculation, we have estimated that we were able to recover almost all cells after switching off the electric field. That means, the sum of the particle concentration increase in the outlet flow after switching off the field is almost equal to the cumulated concentration decrease during the time the field is switched on.

It is well-known that particle retention using dielectrophoresis has a negative effect on cell viability caused by the exposure to the high electric fields^23,46^. We have tested the viability of the released cells using a Thermo Fisher Scientific BacLight LIVE/DEAD cell assay together with an epifluorescence microscope. Viability was determined for the released cells from two experiments performed at Δ*V* = 150 V_RMS_ and *Q* = 1 mLmin^−1^ and at Δ*V* = 300 V_RMS_ and *Q* = 6 mLmin^−1^. Both data points give approximately the same *η*. In both cases, the cell viability of the released cells was very high, above 95% (see supplementary information, Sec. 7, for more details).

## Conclusions

The two classical approaches for DEP-based particle separation, electrode-based and electrode-less DEP, show high accuracy and versatility, but are not able to process larger amounts of liquid as required for many bio-analytical analyses (for example in the detection of circulating cancer cells^3^) or in industrial processes like scrap recovery.

Dielectrophoretic filtration is an electrode-less process that was reported several times in the literature but never elucidated in detail. In principle, since channel size and electrode distance are decoupled, it could be easily scaled up. In this manuscript, we explored the process further. Firstly, we simplified the porous medium by an array of insulating posts. Such arrays in microfluidic chips were readily produced using standard polymer replica methods. We used simulation methods to determine the separation efficiency of such DEP model filter chips and verified key results with experiments. The simulations were able to predict the experiments with sufficient precision, except for low post spacing (*d* ≤ 60 μm) and at low applied voltages in combination with high throughputs (Fig. 2).

Using the simulation we were able to derive straight-forward design rules for model DEP filters. We showed that the separation efficiency *η* is a straight-forward function of $\bar{x}={({\rm{\Delta }}V)}^{2}{d}_{{\rm{P}}}^{2}{Q}^{-1}{h}_{{\rm{S}}}^{-1}{\rm{Re}}[K]$, a variable that includes most of the parameters that influence the separation. This is valid for constant *d*, but the required proportionality constant could easily be found for all post spacings *d*. We showed that this relationship holds for experimental model particles (polystyrene). We used only a single fitting parameter which is the PS particles’ relative polarizability, Re[*K*]. Since the resulting Re[*K*] = 0.52 falls well within reasonable range, we consider the fit to be remarkable. We could further show that the relationship is equally true for real separation systems by separating baker’s yeast from KCl solution.

While the resulting model filter experiments (using microfluidic devices) are attractive to characterize the positive DEP separation mechanics, DEP filtration in porous media bridges the throughput gap. We were able to show that we can easily transfer the results from model filter channels to real macroscopic separation systems. We separated yeast cells using a filter setup (*A* = 2 cm^2^, *d*_50,3_ = 230 μm, porosity 83%) at throughputs *Q* = 1–9 mLmin^−1^. We found a very similar parameter dependency compared to the microchannels, indicating that the separation mechanisms are equal in both setups.

We can now easily describe and observe the separation dynamics in transparent microchannels. At the same time, we have a large-scale setup that operates on the same principles, but at a much higher throughput. The results indicate the possibility for high-throughput DEP particle retention and experiments to show experimentally the degree of selectivity that can be obtained are under way. Consequently, we expect such a process to be capable of solving existing separation problems, such as the detection of cancer cells from whole blood or the recovery of precious metal from a mixture of waste.

## Methods

In this manuscript we performed simulation and experiments using two different experimental setups.

### Microfluidic device design and fabrication

The procedure for designing and producing microfluidic devices with polydimethylsiloxane (Sylgard 184, Dow Corning Corporation) is well established^47^. First, a master mold is created using photo lithography with silica wafers and SU8. To do so, a vector graphic of a two-dimensional mask was created using AutoCAD (Autodesk). Foto masks were produced (CAD/Art Services Inc.) with a resolution of 20000 dpi. A single dot has thus a diameter of 1.27 μm but the smallest feature size is limited to 8 dots or 10 μm according to CAD/Art Services. Schematics of the microfluidic array are given in Fig. 1. In total, seven different designs were produced, with post diameter *h*_S_ and spacing *d* combinations, {*h*_S_, *d*} = {262 μm, 38 μm}, {262 μm, 66.4 μm}, {262 μm, 100 μm}, {262 μm, 130 μm}, {262 μm, 162 μm}, {390 μm, 100 μm}, {520 μm, 100 μm} (see supplementary information, Fig. S6, for an exact drawing of the channels). The periodic array represents the porous medium and has a length of 8.5 mm. In front and at the end of the channel half-elliptic posts are employed to avoid clogging of the channel due to any dust that might have been entrapped in the channel during production. The open channel between inlet and outlet and the array contains triangular support structures to prevent the channel from collapsing. The microfluidic device design and fabrication from this work is similar to the one used by Kawale *et al*.^48^ for flow visualization of polymer solutions in model porous media.

Before the soft lithography step the SU8 negative was preconditioned with Trichloro(1*H*, 1*H*, 2*H*, 2*H*-perfluorooctyl)silane in an evacuated desiccator for a minimum duration of 60 min. That allows an easier peel-off after curing. Sylgard 184 was mixed in a ratio of 10:1 (polymer to curing agent), degassed, and poured on the preconditioned SU8/Silica negative. Channels were cured at 140 °C for 15 min. After peeling off the PDMS designs from the wafer, three chips were diced according to the size of a microscope slide. Holes for the connection of the PTFE tubing (ID/OD 300 μm/1.6 mm, Kinesis) were punched with 1.5 mm diameter biopsy punches (World Precision Instruments Germany GmbH). Two holes for the electrodes (500 μm platinum wire) were punched with 0.5 mm diameter biopsy punches (also World Precision Instruments). PDMS-covered glass slides were prepared by spin coating isopropanol cleaned microscope slides with uncured PDMS at 3000 RPM for a duration of 1:30 minutes. The glass slides were subsequently cured at 70 °C for 60 min. The PDMS-covered glass slides and the diced PDMS chips were cleaned 5–6 times using Scotch magic tape (3 M Company) and ethanol. Both sides of the channel were activated by exposing them to a low-pressure air plasma for 1:30 minutes. They were subsequently bonded by gently pressing them together. The channels in which all internal sides were PDMS had a final height of 120–130 μm (depending on the wafer used) and a width of 2.8 or 3.2 mm, depending on the employed geometry. All flow rates in the manuscript are given for a channel of width 2.8 mm, flow rates for channels with *W* = 3.2 mm were multiplied by a proportionality factor to yield an equal superficial velocity.

### Experimental setup

#### Microfluidic devices

A sinusoidal ac electric field was applied across the array using two 500 μm platinum wires connected to an ac high-voltage source (Trek PZD2000A in combination with a Rigol DG4000 abritrary waveform generator). Fluid was put into the channel using a syringe pump (kdScientific Legato 270 loaded with 3 mL PlastiPak disposable syringes) via a PTFE tubing (ID/OD 300 μm/1.6 mm). To calculate separation efficiencies, particle flux into and out of the post array has been observed using an upright epifluorescence microscope (Carl Zeiss Axio Scope.A1 Vario equipped with a 5 × EC Epiplan objective glass) and recorded using a Lumenera Infinity 3S-1URM camera (exposure time 15 ms) and the 65 HE filter set (Carl Zeiss, BP 475/30, FT 495, BP 550/100).

#### Filtration setup

A schematic of the setup is shown in Fig. 4a and a photograph in Fig. 4b. The volumetric flow through the filtration cell was controlled by a peristaltic pump REGLO Analog (Ismatec).

The filtration setup consisted of a PTFE body, a PMMA cover, and two stainless steel plate electrodes that were aligned perpendicular to the filtrate flow with a separation distance of 8 mm. A replaceable ceramic filter with cross-section of 7 × 29 mm (*A* = 2 cm^2^) and length of 18 mm was placed between the plate electrodes. The filter were produced from 45% alumina and 55% sintered mullite using a foaming technique. They had a porosity of 83% and a median pore diameter of 130 μm and a median window diameter of 44 μm. The pore diameter was determined using the algorithm of Rabbani *et al*.^49^ (which is a Matlab implementation of the watershed segmentation algorithm) from height profile images (observed area of 41 mm^2^) taken with a laser scanning microscope (Keyence VK-X200). The window diameter was determined from manual counting performed on the same image. A histogram of the pore size distributions as well as a sectional view of the filter can be found in the supplementary information (Sec. 5). Their flow resistence was 55 kPasm^−2^ determined according to DIN EN 29053:1993. A sinusoidal ac voltage of 150 V_RMS_ and 300 V_RMS_ was applied across the filter using a function generator HM8131-2 (Hameg) and a power amplifier PZD700A Dual channel (Trek).

Two different types of electrodes (wire and plate) where used in the two different cases (microchannel and macroscopic filter, resp.). Our simulations show, however, that generation of an electric field with small Platinum wires generates sufficiently homogeneous superficial (without separation matrix) electric field (just as we assume it is the case with using plate electrodes).

### Particle suspensions

Two kinds of particle suspension were used in this study, polystyrene microbead suspension and yeast cell suspension. Standard monodisperse polystyrene particles (Fluoresbrite, diameter 1 μm, COOH-functionalized, YG, ex/em 441/487, roughly FITC) were purchased from Polysciences Europe and diluted to a final concentration of 2 × 10^5^–8.5 × 10^5^ particles mL^−1^, depending on the flow rate employed. The electric conductivity has been adjusted using KCl to a value of 3.7 × 10^−4^ Sm^−1^. A homeopathic amount of Tween 20 (0.05 vol%) was added to the solution to avoid particle adsorption on the PDMS surface.

Culturing yeast cells is straight-forward and well-described: A block baker’s yeast was purchased from the supermarket. A small amount of the block was transferred to a culture flask and incubated in YPD culture medium (yeast extract peptone dextrose) for approximately 18 hours. Yeast cells were harvested without paying attention the log-phase of the culture. The cells in culture medium were separated from the culture medium by centrifugation for 10 min at 2500 rpm. The yeast pellet was subsequently resuspended in PBS (phosphate buffered saline), diluted to an optical density at 600 nm of 0.5, and labeled using a standard LIVE/DEAD BacLight labeling kit as commercially available from Thermo Fisher Scientific (contains the proprietary SYTO 9 and propidium iodide stains). An amount of 1 μL SYTO 9 and 2 μL propidium iodide was added per mL of cell suspension. The mixture was incubated for 15 mn in the dark, subsequently centrifuged, and resuspended in an equal amount of KCl suspension of conductivity 2.7 × 10^−4^ Sm^−1^. Fluorescence microscopy using green and red filter sets revealed that in all cases, more than 95% of all cells only show green fluorescence, indicating that almost all cells (95–100%) were viable prior to the experiment. For microfluidic experiments, yeast cells were diluted to a final concentration of approximately 1 × 10^5^ cells mL^−1^. For experiments using the macroscopic filtration setup, cells were diluted to a final concentration of approximately 5 × 10^4^ cells mL^−1^.

### Experimental procedure

#### Microfluidic experiments

Each data set was obtained using four different channels (with the same geometry). Due to hydrophobic recovery (due to aging of the PDMS), channels were flushed before each experiment with ethanol to achieve full wetting of the inner surface. Then, particle suspension (PS suspension or yeast cell suspension) was filled in the channel; before first use, each channel was flushed for at least 10 min with particle suspension. For each voltage and flow rate combination, three videos where recorded at the inlet and outlet, for a duration of 70 sec and 100–160 sec each. The different recording times at the outlet to account for different *Q* and *d*, generally we recorded videos up until we reached a point where the particle flux became time-independent. After each video, the flow was increased to 30 mLh^−1^ for 15 sec to flush all trapped particles or cells out of the channel. Particle fluxes have been calculated from the recorded videos using an in-house MATLAB program that can detect particle centers on dark background due to gray value thresholding (based on DIPimage library, http://www.diplib.org/). The Hungarian Linker algorithm (https://de.mathworks.com/matlabcentral/fileexchange/34040-simple-tracker) is used to create particle tracks. The separation efficiency was calculated as *η* = (*ṅ*_in_ − *ṅ*_out_)/*ṅ*_in_ with the particle flux into and out of the channel, *ṅ*_in_ and *ṅ*_out_, resp. Both fluxes were averaged over the last 20 sec of each recorded video. Since *ṅ*_in_ decreases slightly after turning on the field (due unwanted trapping at the electrode), outlet videos were recorded for a longer time than inlet videos. The time at the outlet until a time-independent *ṅ*_out_ is achieved depends on *d* and *Q* and is somewhere between 60–120 sec. Separation efficiency is calculated by averaging over a total of 16 values, that is, 4 channels with 3 inlet-outlet video pairs each.

We applied an electric field of frequency 30 kHz to separate PS particle and 15 kHz for yeast cell suspension. We could only use *Q* > 0.5 mLh^−1^ in the case of yeast cells because otherwise, the cells would settle too quickly in the channel due to gravity. All experiments were carried out at room temperature.

#### Filtration experiments

Filtration efficiency was determined by using a fluorescence spectroscopy Fluoromax-4 (Horiba) and a quartz flow-through cuvette 176.762-QS (Hellma). This allowed us to direct the filtered suspension directly through the spectrometer after passing the setup. By this we can detect a temporally resolved fluorescence intensity signal. For the low particle concentrations used, the fluorescence intensity, emitted from the stained yeast cell suspension, linearly increases with the particle concentration in the suspension and thus allowed to determine the yeast cell concentration with high accuracy. The fluorescence labelled yeast cells were excited at a wavelength of 485 nm. Emission was detected (perpendicularly to the excitation) at a wavelength of 502 nm (this matches the excitation and emission maxima of SYTO 9). The trapping efficiency *η* was determined with *η* = 1−CPS_DEP_/CPS_0_, with the fluorescence intensities (counts per second, CPS) of the suspension after passing through the filtration cell with applied and without electric field, CPS_DEP_ and CPS_0_, respectively. In each experiment, we waited until the intensity signal was stationary to be sure that delayed effects like, for instance, flow mixing are not affecting the results. After each filtration experiment, we switched off the field and waited until all trapped cells exited the channel (i. e., until the intensity became time-independent). Then, we set the setup to the next set of parameters and conducted the next experiment. All experiments were carried out at room temperature.

### Simulation

**Figure 5:**
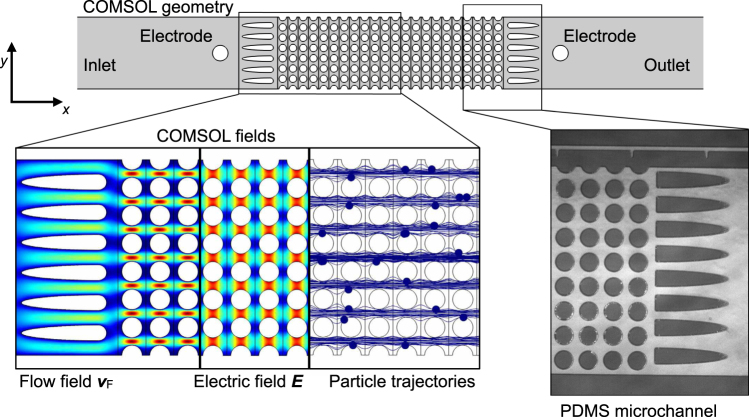
Sketch of the geometry as modeled in COMSOL. Microchannels are two-dimensional and show a similar geometry compared to the actual microchannels. The insets show surface plots of ${\overrightarrow{v}}_{{\rm{F}}}$ (the fluidic velocity due to the applied pressure difference), $\overrightarrow{E}$ (the electric field due to the applied potential drop Δ*V*), and calculated particle trajectories. On the right is, for comparison, a bright-field microscopy image of an actual PDMS channel.

The microchannels (Fig. 5) were modeled using COMSOL (COMSOL Multiphysics 5.1). The channel differed in width (2.8 mm for the experimental channel and approx. 4 mm for the simulated channel); the flow rate was adjusted in the simulation to account for this difference, so that both channels experience the same superficial velocity. The velocity distribution, ${\overrightarrow{v}}_{{\rm{F}}}$, was calculated using the Creeping Flow module that solves Stokes Equations. The velocity at the inlet was prescribed so that the calculated flow rate *Q* matches that of the experiments. Constant pressure of *p* = 0 was prescribed at the outlet and no-slip boundary conditions were prescribed on all solid sides.

The electric field was obtained by solving Laplace’s equation (Electrostatics module). The potential of ±Δ*V*/2 was prescribed at the electrodes and Neumann boundary conditions were prescribed on all solid surfaces as well as on the inlet and outlet.

We assumed that the particles always move at their terminal velocity. We thus rendered the particles massless and obtained their trajectories by solving their equation of motion under consideration of dielectrophoresis and fluidic drag (using the Particle Tracing module):

3 $$\frac{\partial {\overrightarrow{x}}_{i}(t)}{\partial t}={\overrightarrow{v}}_{{\rm{F}}}({\overrightarrow{x}}_{i})+2{\mu }_{{\rm{DEP}}}\nabla {|{\overrightarrow{E}}_{{\rm{RMS}}}({\overrightarrow{x}}_{i})|}^{2},$$

with position vector ${\overrightarrow{x}}_{i}$ of the *i*-th particle (*i*∈[1,*n*], *n* is the total number of investigated particles) and the dielectrophoretic mobility ${\mu }_{{\rm{D}}{\rm{E}}{\rm{P}}}=({d}_{{\rm{P}}}^{2}{\varepsilon }_{{\rm{m}}}{\rm{R}}{\rm{e}}[K])/24{\mu }_{{\rm{F}}}$. Here, *μ*_F_ is the dynamic viscosity of water, *μ*_F_ = 1 × 10^−3^ Pas (assuming room temperature, ~23 °C). For each simulated dataset, *n* = 250 particles were simulated that have been randomly introduced over the entire microchannel depth on a straight vertical line between the electrode and the dust blockers. That means, particles were added to the fluid stream (initialized) well after the inlet to significantly reduce simulation time (the unimportant part of the trajectory from the inlet to the post array is skipped). Each calculation was performed three times, so a total of 750 particle trajectories with random start positions were simulated per data point. The simulation is two-dimensional and assumes particles of negligible volume, thus only traces particle centers. The acting forces due to DEP and drag were calculated assuming values at the particle center. As before, the separation efficiency is calculated according to *η* = (*ṅ*_in_ − *ṅ*_out_)/*ṅ*_in_. Mesh independence was investigated by changing the maximum element size between the posts by one order of magnitude in each direction. The separation efficiency (which is the target value) was not affected by that change, indicating mesh independence of the solution. The same holds for the number of simulated particles, the number of repetitions, and the relative tolerance of the time-dependent solver in COMSOL when these values were changed by significant amounts.

### Clausius-Mossotti factor

According to Eq. (1), the DEP force scales linearly with the real part of the Clausius-Mossotti factor Re[*K*]. Albeit there are some reports on determination of the particle’s polarizability^39,50,51^ for our specific particles (COOH-modified PS particles from Polyscience and cultured yeast cells), we do not know this factor a priori.

In order to obtain a a match between the simulated data using polystyrene particles and the experiments we have performed simulations assuming a variety of Re[*K*] values. Best matches for Fig. 2 (visually) were achieved when a value of Re[*K*] = 0.52 was assumed in the simulation. Consequently, we assume that the real polystyrene model particles have a value of Re[*K*] = 0.52 in the medium conductivity of *σ*_m_ = 2.7 × 10^−4^ Sm^−1^. For 1 μm particles, this is a very reasonable assumption when compared to the literature data^39,50,51^. After obtaining the fit in Fig. 3, we can easily determine the Yeast cells’ Re[*K*] value: The experimentally determined *η* values for Yeast (stars) will move toward the left or right in the graph with a change of Re[*K*]. A value of Re[*K*] = 0.18 gives a good match between the fit from simulation and the experimentally determined *η* for yeast cells.

### Data Availability

The datasets generated during and/or analyzed during the current study are available from the corresponding author on reasonable request.

## Electronic supplementary material

Supplementary Information

Supplementary Movie S1
