# Supplementary material for: Bridging the scales in high-throughput dielectrophoretic (bio-)particle separation in porous media
Source: Sci Rep. 2018 Jul 11;8:10480. doi: 10.1038/s41598-018-28735-w (PMC6041321; doi:10.1038/s41598-018-28735-w)
Supplement: Supplementary file 1 — Supplementary Information [file 41598_2018_28735_MOESM1_ESM.pdf]

Bridging the scales in high-throughput dielectrophoretic (bio-)particle separation in porous media

Supplementary information

Georg R. Pesch, Malte Lorenz, Shaurya Sachdev, Samir Salameh, Fei Du, Michael Baune, Pouyan E. Boukany, and Jorg Thöming

1. Additional data to Figure 2

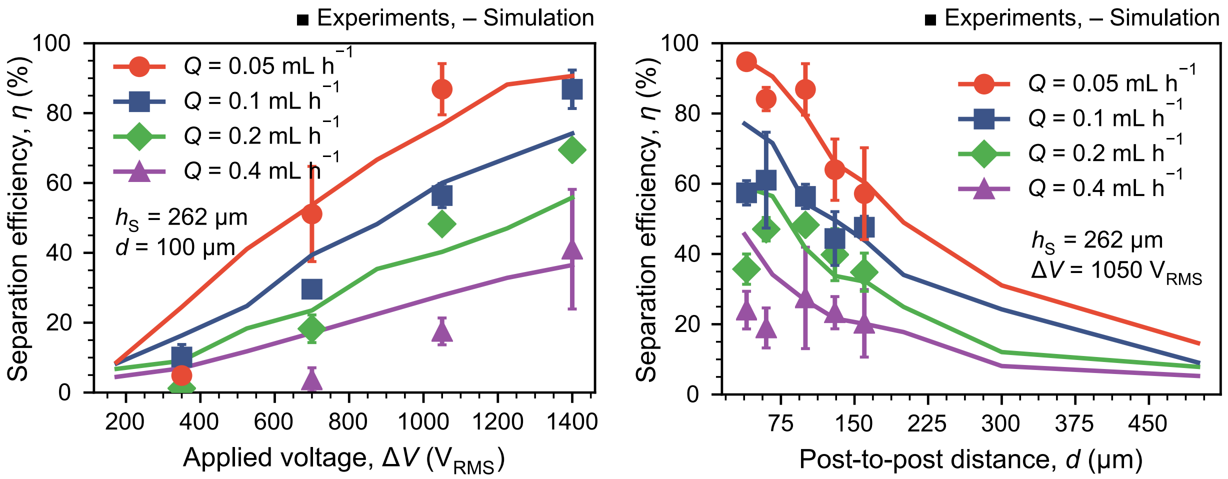

Figure S 1: Additional data to Figure 2. Left panel shows Fig. 2 (a) with an additional data set at $Q = 0.4$ ml h^{-1}. Right panel shows Fig. 2 (b) with the simulated separation efficiencies up to 500 μm.

2. Quantification of the finite size effect

The experimentally determined separation efficiency shows increasing deviation from the simulations with decreasing post-to-post spacing. In other words, the simulation predicts a monotonically increasing separation efficiency with decreasing spacing, whereas the experiments suggest an ideal post-to-post spacing. We assume this deviation is because the simulation neglects the particles' finite size. Since the simulation only calculates the trajectories of volume-less particle centers, particles will always be immobilized directly on the posts' surface. In reality, however, when the particle is immobilized its center will be one radius away from the post's surface. The force balance (i.e., DEP force vs. drag force) will be substantially different in both cases (see Fig. S 2). A balance between drag and DEP force (Fig. S 3, left) reveals that in the real case, the DEP force is not always strong enough in order to keep the particle irreversibly on the post's surface. Especially at low values of θ, the drag force is larger than the DEP force which would forbid trapping at that location. This is not considered in the simulation. The gravity of this effect can be expressed in terms of the intersection angle θ_0 which gives the point on the posts surface at which the DEP force becomes larger than the drag force.

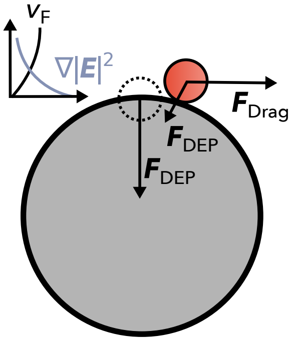

Figure S 2: DEP and drag force directly on the surface of the particle (dotted particle outline, as it occurs in the simulation) and one radius away from the post (red particle, is it occurs in reality). Because of the parabolic flow profile and the no-slip boundary condition, the drag force is zero directly on the post's surface. With increasing distance from the post, the force balance shifts from DEP to drag.

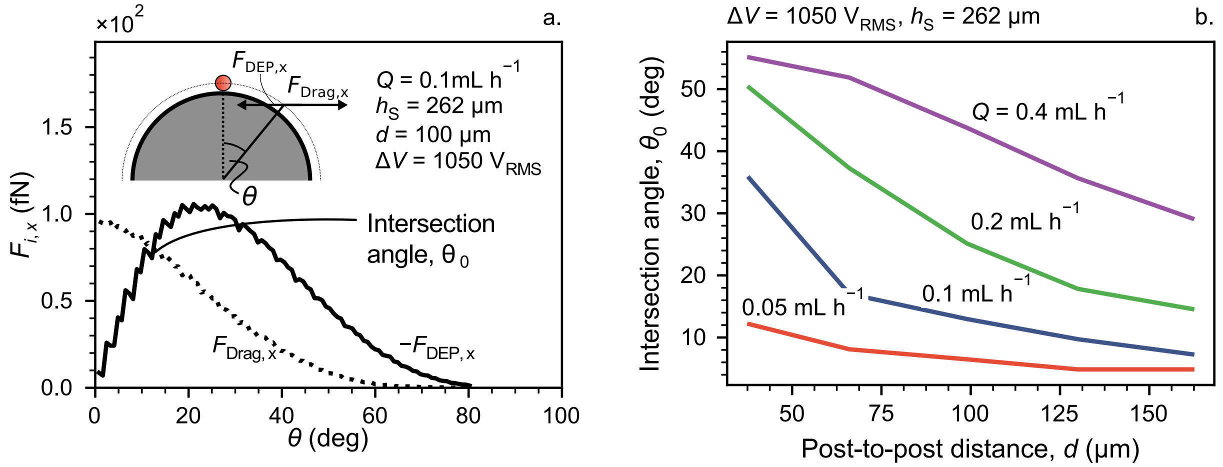

Figure S 3: It is possible to quantify the finite size effect by comparing the DEP force against the flow direction -$F_{DEP,x}$ with the drag force that opposes the particle trapping $F_{Drag,x}$ on all locations the surface of the post (actually, one particle radius away from it) as expressed by the angle θ (a). At $\theta = 0$, thus directly at the pore throat, the fluid velocity is highest (due to the constriction). At the same time, $F_{DEP,x} = 0$, since all DEP force only points in y direction. With increasing θ, the $F_{DEP,x}$ increases while simultaneous $F_{Drag,x}$ decreases (because the pore widens due to circular post design). The angle $\theta = \theta_0$ where -$F_{DEP,x}$ becomes larger than $F_{Drag,x}$ is the intersection angle. At values $\theta < \theta_0$, albeit being predicted by the simulations, no trapping can occur since the drag force would wash particles away. Thus, a large value indicates a strong overprediction of the simulated separation efficiency compared to the experiments. As expected, θ_0 is low for all d at Q = 0.05mL h^{-1}. It is increasing with decreasing d and increasing Q since both parameter variations would cause a shift of the force balance toward higher drag forces.

It is possible to shift this force balance toward DEP by varying the cross-sectional aspect ratio (AR, width-to-height ration of the cross section) of the posts (that is, skewing the post's cross section so that the longer axis would be aligned perpendicular to the applied field). This will cause substantially higher DEP forces close to the immobilization points as it is obvious from a decreasing value of θ_0 (Fig. S 4, left). This decrease is more pronounced for Q = 0.1 mL h^{-1} than for Q = 0.2 mL h^{-1}. This should cause a decreasing deviation between the experiments and the simulation for AR > 1. From Fig. S 4 (right), it is obvious that both, simulated and experimentally determined separation efficiency increase with increasing aspect ratio (due to the larger overall DEP forces). Nevertheless, it is possible to observe that the deviation between experiment and simulation decreases with increasing aspect ratio (due to the shift in the force balance from drag to DEP and thus the decreasing intersection angle θ_0). As an example, the deviation is over 15 % for AR = 1 and Q = 0.1 mL h^{-1} but less than 5 % at AR = 1.6. This directly supports the theory that the neglect of the particles finite size causes the overprediction of the simulation results. We assume that the finite size effect also depends on the particle size. With decreasing particle size the ideal spacing should shift towards smaller values.

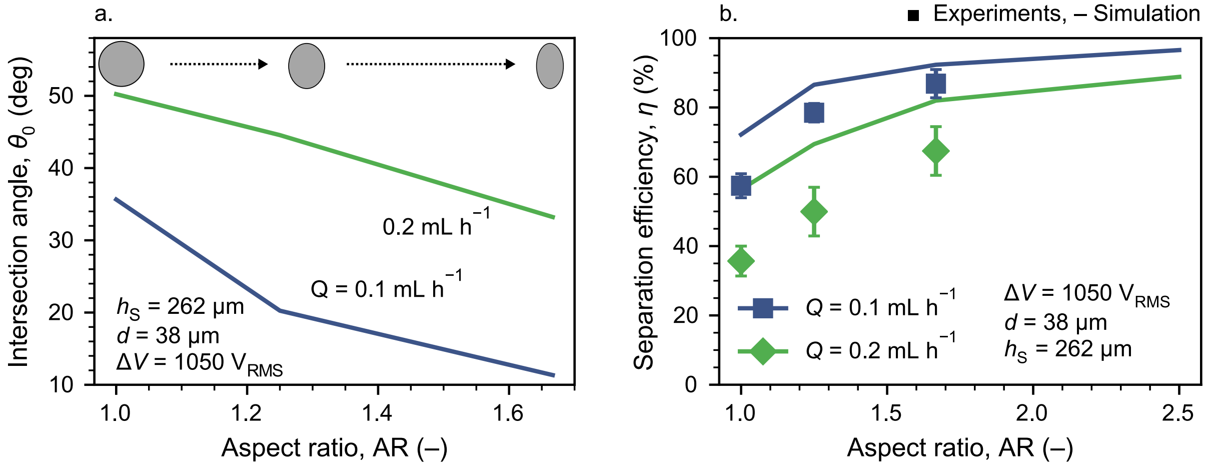

Figure S 4: Intersection angle as a function of the cross-sectional aspect ratio (width-to-height ratio) (left) and (right) separation efficiency (simulated and experimentally determined) for different aspect ratios.

3. Separation efficiency for different post spacings

Fig. S 5 shows the separation efficiency as a function of $\bar{x} = (\Delta V)^2 d_{\mathrm{P}}^2 Q^{-1} h_{\mathrm{S}}^{-1} \mathrm{Re}[K]$ (as Fig. 3 a from the main document but for different post spacings *d*). The figure also includes fits of type $\eta = 100\%(1 - \exp{(\bar{x}/C)})$ (Eq. (2) of the main document). Since we only simulated four data points per *d*, the fit is rather poor. We argued that a value of *C* close to 0 indicates a geometry well-suited for particle separation. With increasing magnitude of *C*, the S-shape of the curve (where η switches from 0 to 100%) moves towards higher values of $\bar{x}$ (less efficient separation).

Clearly, decreasing *d* causes an increase in *C*. This is especially evident from Fig. S 5 (right). Here, *C* is shown for all investigated *d* and also a linear fit that links *C* and *d*, i.e. $C = -2.46 \times\ 10^3\ \mathrm{V_{RMS}^2 hm^{-3}} \cdot d$.

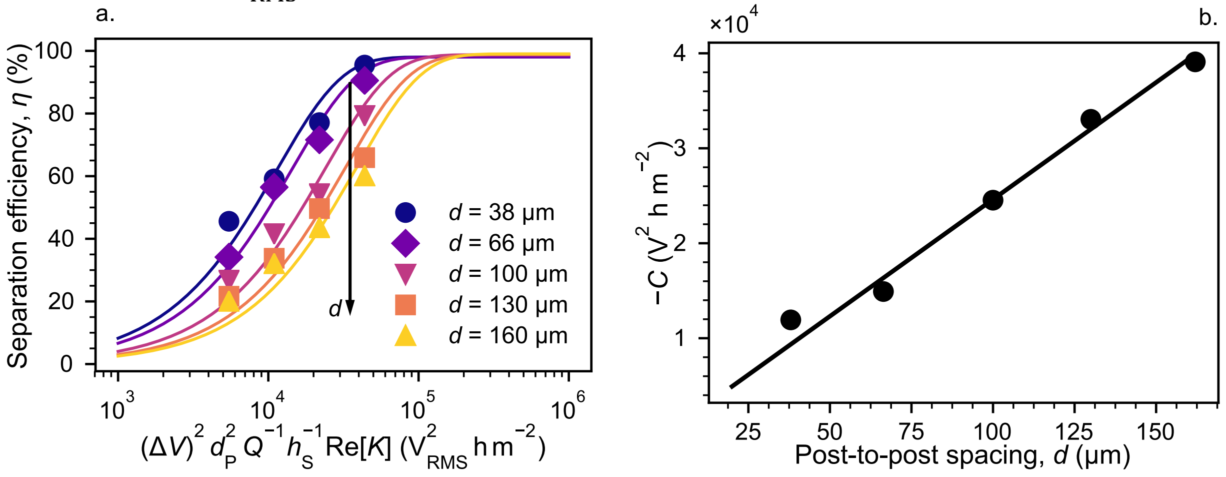

Figure S 5: Separation efficiency η as a function of $\bar{x} = (\Delta V)^2 d_P^2 Q^{-1} h_S^{-1} Re[K]$ for different spacings *d* (left, compare to Fig. 3 a of main document). The right panel shows the constant *C* (Eq. (2) of main document) that relates η with $\bar{x}$ for different *d* as a well as the fit $C = -2.46 \times\ 10^3\ V_{RMS}^2 hm^{-3} \cdot d$ (we refer to this value in the main document).

4. Drawing of the employed microchannels

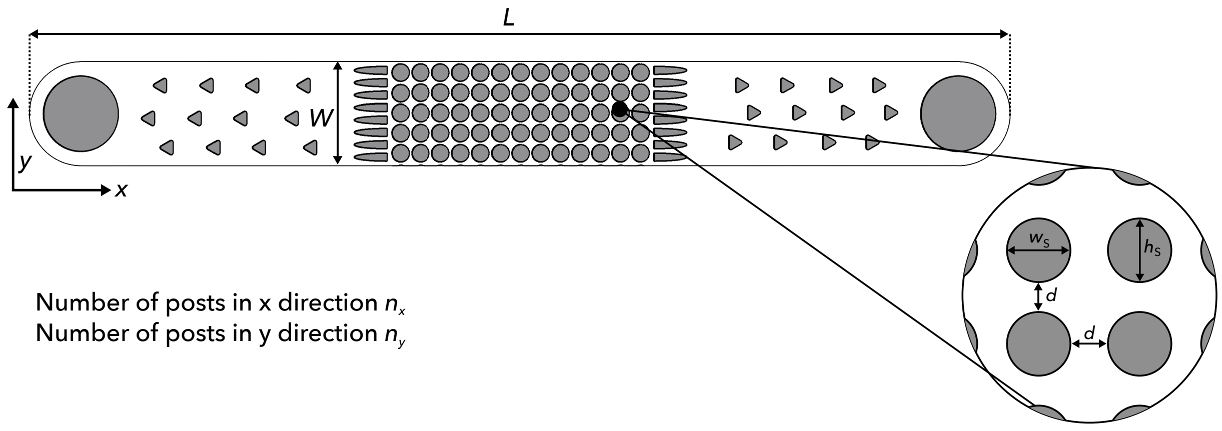

Figure S 6: Sketch and important parameters of the microchannels employed in this study. Sketch was exported from AutoCAD. The parameters can be found in Table S 1.

Table S 1: Parameters of all microchannels used in this study. Some channels differ in height *h* and in their width *W*. All volume flows given in the manuscript are concerning channels with h = 120 μm and W = 2.78 mm. The volume flow of channels with other parameters was multiplied by a factor to obtain an equal superficial velocity.

h_S	w_S	d	W	L	height h	n_x	n_y
μm	μm	μm	mm	mm	μm	–	–
262	262	38	2.78	43.75	120	27	9
262	262	66.4	2.78	43.75	120	25	8.5
262	262	100	2.79	30	130	22	7.5
262	262	130	2.79	30	130	21	7
262	262	162	2.78	43.75	120	20	6.5
360	360	100	3.19	30	130	16	6.5
520	520	100	3.19	30	130	13	5
262	209.6	38	2.79	30	130	33	9
262	157.2	38	2.79	30	130	42	9

5. Characterization of the employed filter

The filter has a volumetric porosity of 83%, a median pore diameter of 130 μm and a median window diameter of 44 μm (both number-based).
The pore diameter was determined using the algorithm of Rabbani et al. (2014) (Ref. 48 main document) (which is a Matlab implementation of the watershed segmentation algorithm) from height profile images (observed area of 41 mm^2) taken with a laser scanning microscope (Keyence VK-X200, Figs. S8 and S9).
The pore window diameter was determined from manual counting on an incident light microscopy image (also Keyence VK-X200, Figs. S10 and S11). The counting was performed by placing 300 ellipses on the image using the software ImageJ and by measuring their resp. half axis.
The filter are produced using a foaming technique and consist of 45 % alumina and 55 % sintered mullite.

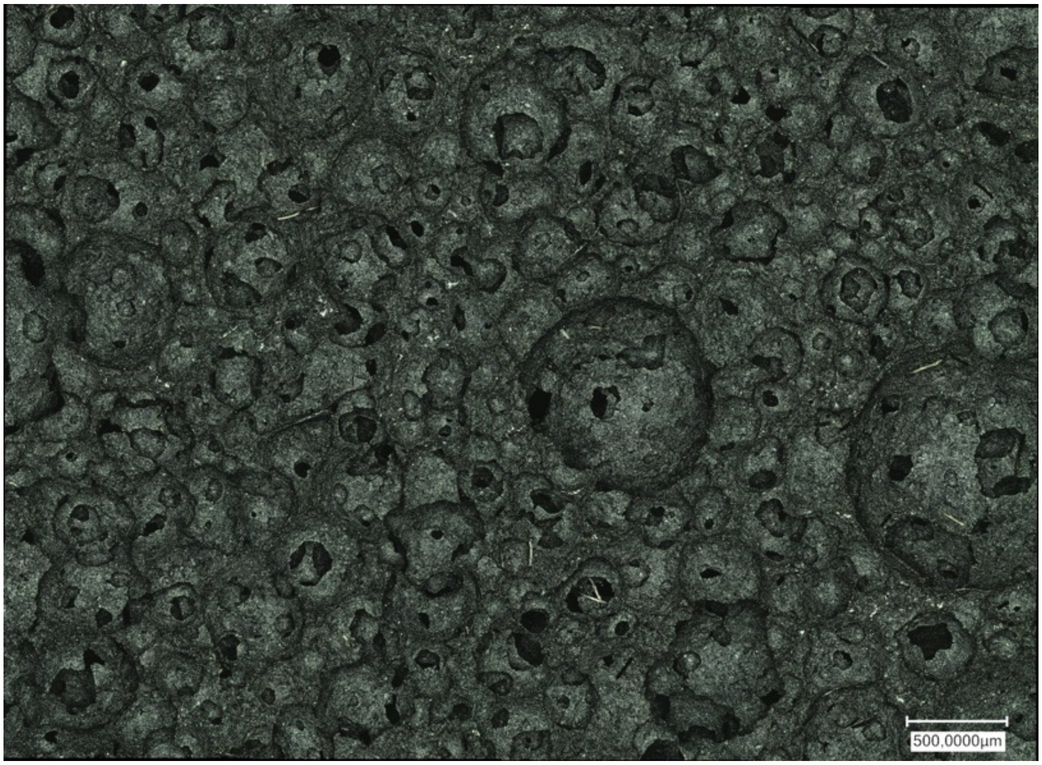

Figure S 7: Sectional view of the ceramic filter.

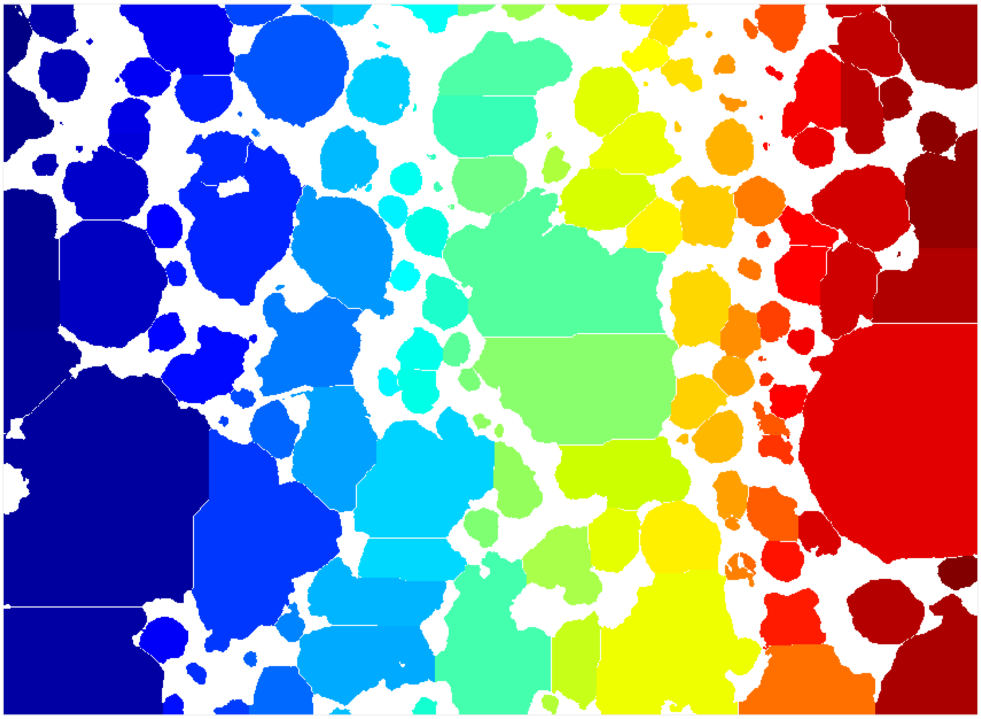
Figure S 8: Segmented pores as detected by the watershed algorithm.

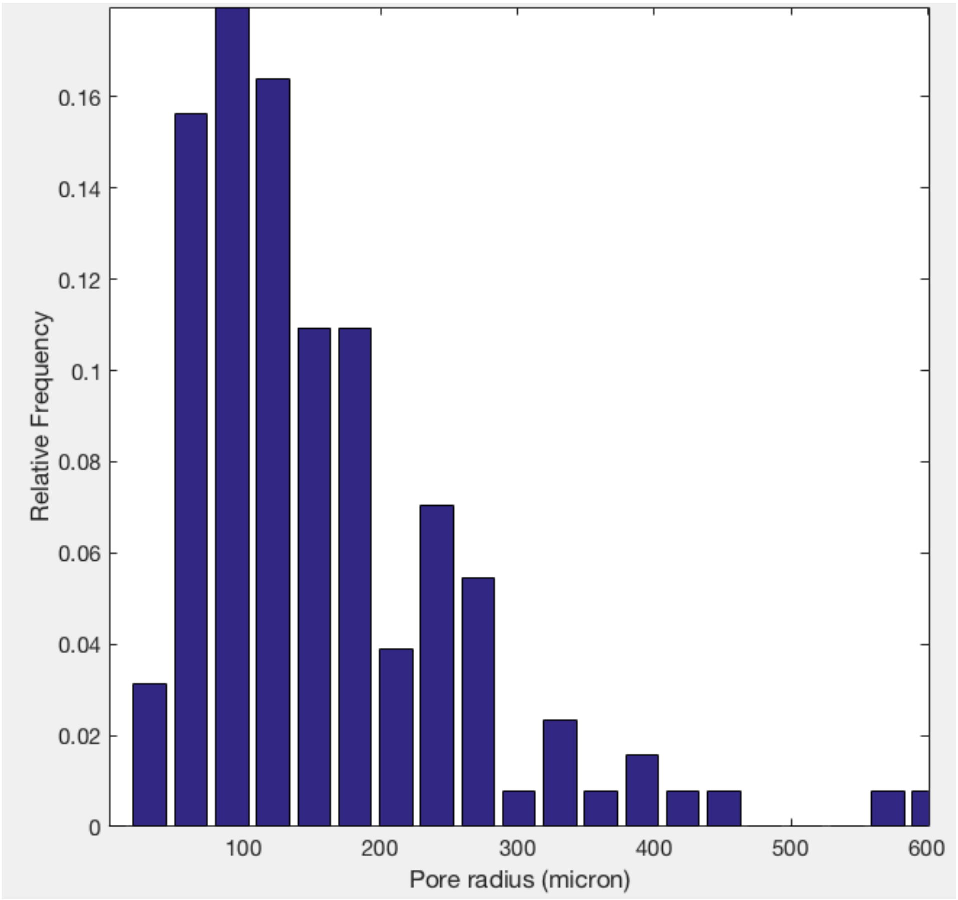

Figure S 9: Histogram of the pore size distribution (as obtained from the watershed algorithm).

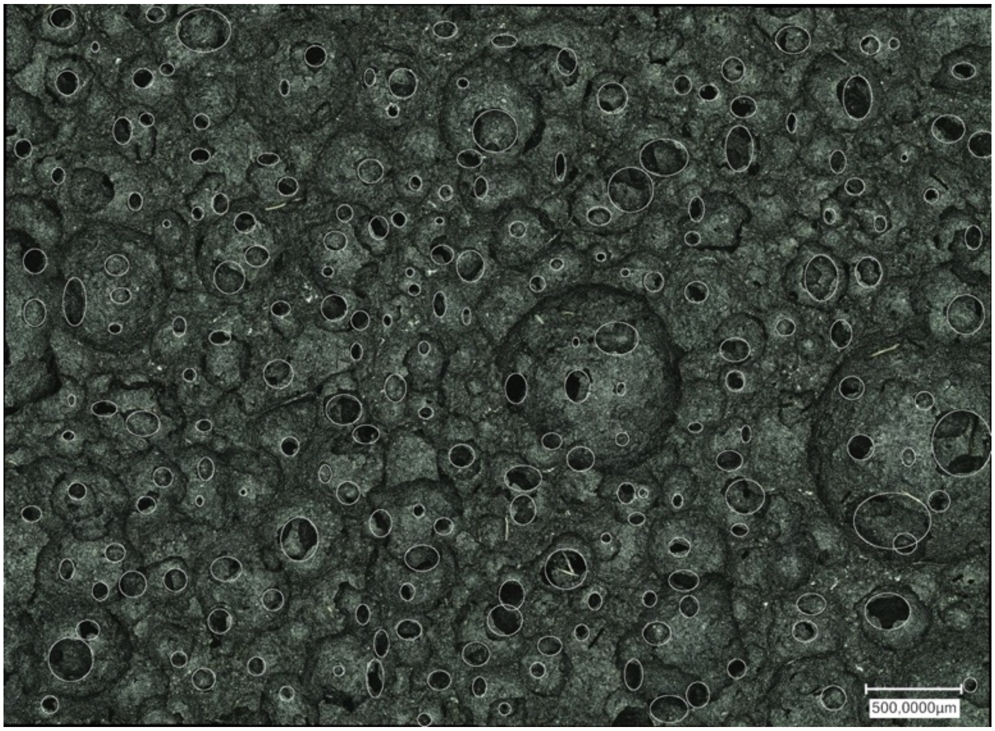

Figure S 10: Manual counting of the pore windows using ImageJ.

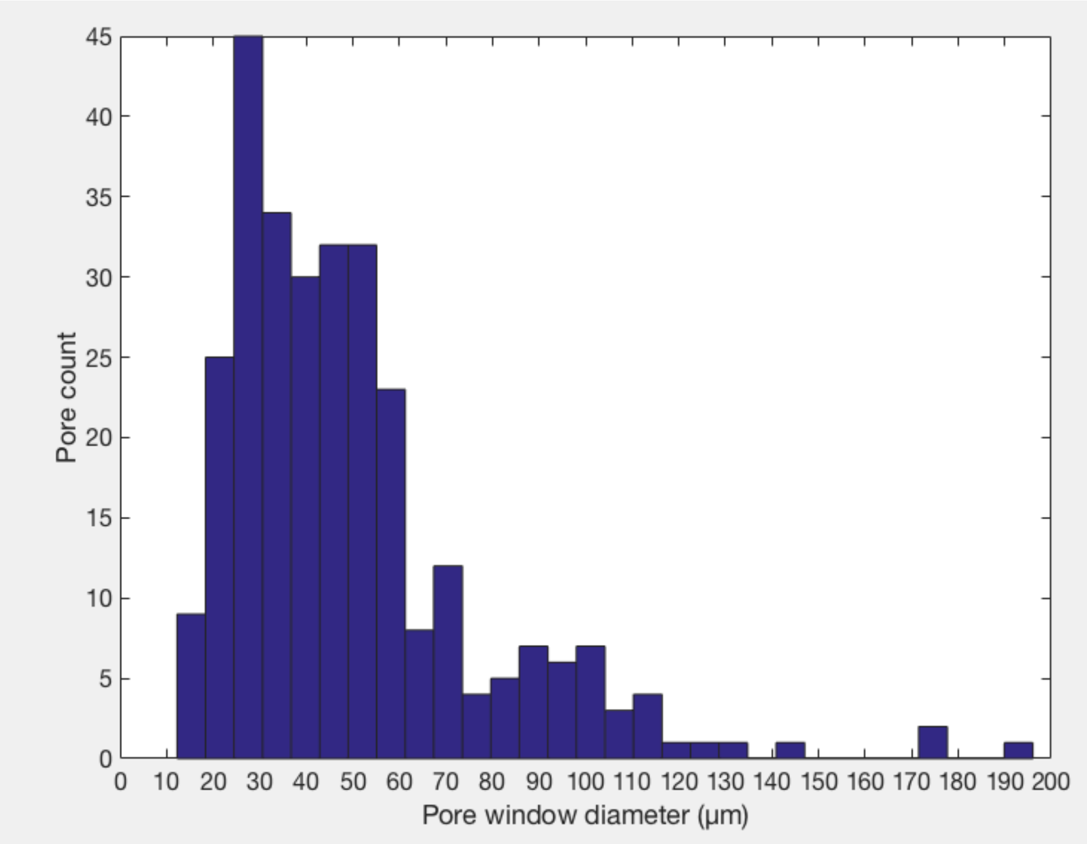

Figure S 11: Histogram of the manual pore window count.

6. Additional Data to Figure 3

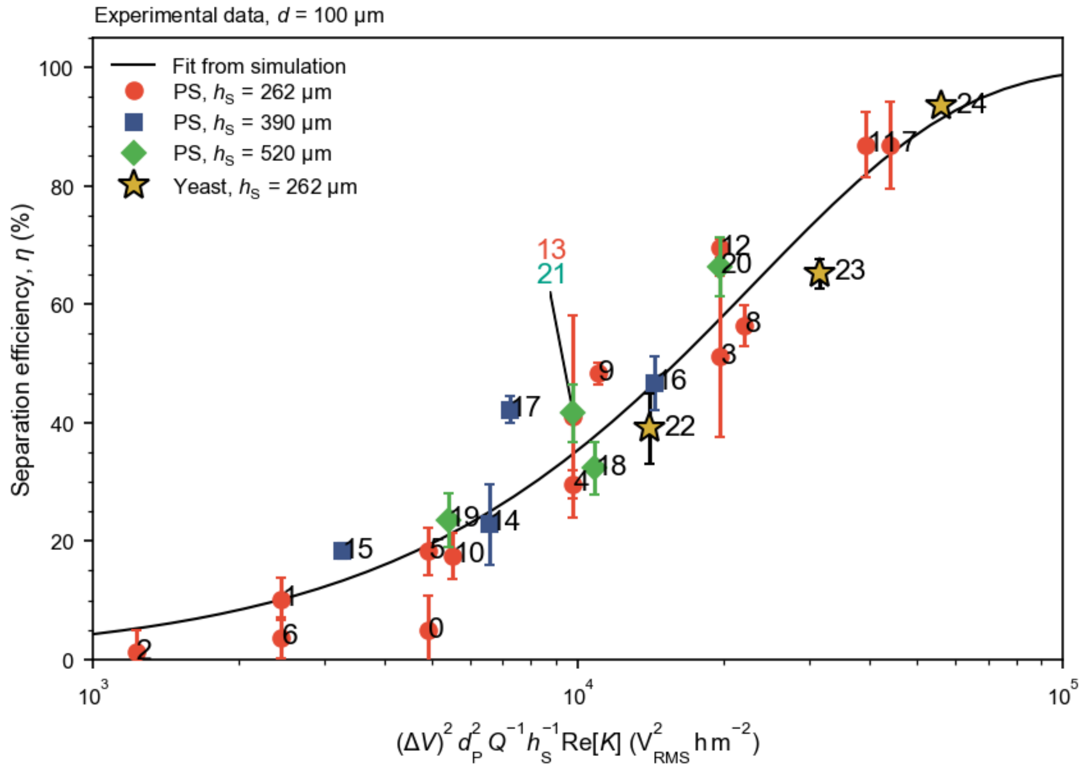

Figure S 12: Additional data to Fig. 3(b). All points are numbered and the corresponding parameters can be found in Table S2.

Table S 2: Parameters for obtaining the data points of Fig. S12.

**#**	**Δ*V* (V)**	***h*S (µm)**	***Q* (mL/h)**	**Sep. Eff.**	**Std. Dev.**
0	350	260.00	0.05	0.049	0.060
1	350	260.00	0.10	0.102	0.036
2	350	260.00	0.20	0.012	0.037
3	700	260.00	0.05	0.511	0.136
4	700	260.00	0.10	0.296	0.024
5	700	260.00	0.20	0.183	0.040
6	700	260.00	0.40	0.036	0.035
7	1050	260.00	0.05	0.869	0.073
8	1050	260.00	0.10	0.564	0.035
9	1050	260.00	0.20	0.483	0.019
10	1050	260.00	0.40	0.175	0.038
11	1400	260.00	0.10	0.868	0.055
12	1400	260.00	0.20	0.695	0.016
13	1400	260.00	0.40	0.410	0.171
14	700	390.00	0.10	0.228	0.068
15	700	390.00	0.20	0.183	0.013

16	1040	390.00	0.10	0.467	0.046
17	1040	390.00	0.20	0.422	0.022
18	1040	520.00	0.10	0.323	0.045
19	1040	520.00	0.20	0.235	0.046
20	1400	520.00	0.10	0.663	0.049
21	1400	520.00	0.20	0.416	0.049
22	700	260.00	0.60	0.390	0.059
23	1050	260.00	0.60	0.652	0.025
24	1400	260.00	0.60	0.934	0.005

7. Cell viability before and after separation experiments

The cell viability in all cases was determined using the BacLight LIVE/DEAD viability assay from Thermo Fisher Scientific. This assay uses SYTO 9 (green fluorescent) as cell stain for all cells and propidium iodide (PI, red fluorescent) stain for dead cells. Hence, after labeling, all cells are labeled with green fluorescent color whereas, additionally, only dead cells are labeled by red fluorescent color.

Before the experiment, we use 3 μL of a 1 to 1 mixture of both components, SYTO 9 and PI (as obtained from the supplier) per 1 mL of cell suspension (concentration of the cell suspension is roughly 5×10^6 cells/mL). After 20 minutes incubation the cells are washed one time using pure water via centrifugation for 10 minutes (to remove all residual cell stain in suspension).

Since all residual dye is washed off, after experiments we collect 10 mL of the effluent (that we want to determine) and add another 3 μL of a 1 to 1 mixture of both components. This is again incubated in the dark for 20 minutes. Since the cell concentration is too low to find enough cells for a quantitative analysis using a fluorescence microscope together with microscope slide and a cover slip, we filled a spare PDMS channel with the stained cell suspension. This allows to find a sufficient number of cells on one image for quantitative analysis. At this stage we do not remove the residual dye because the concentration of cells is too low for centrifugation (no pellet is formed).

We performed viability analysis of the released cells in the filtration experiments for two data points of Fig. 4C, namely, 300 V and 6 mL/min flow rate and 150 V and 1 mL/min flow rate. Both data points have approximately the same separation efficiency of ~80 %. In both cases the cell viability of the released cells is very high, almost 100 % (see Figs. S14 and S15).

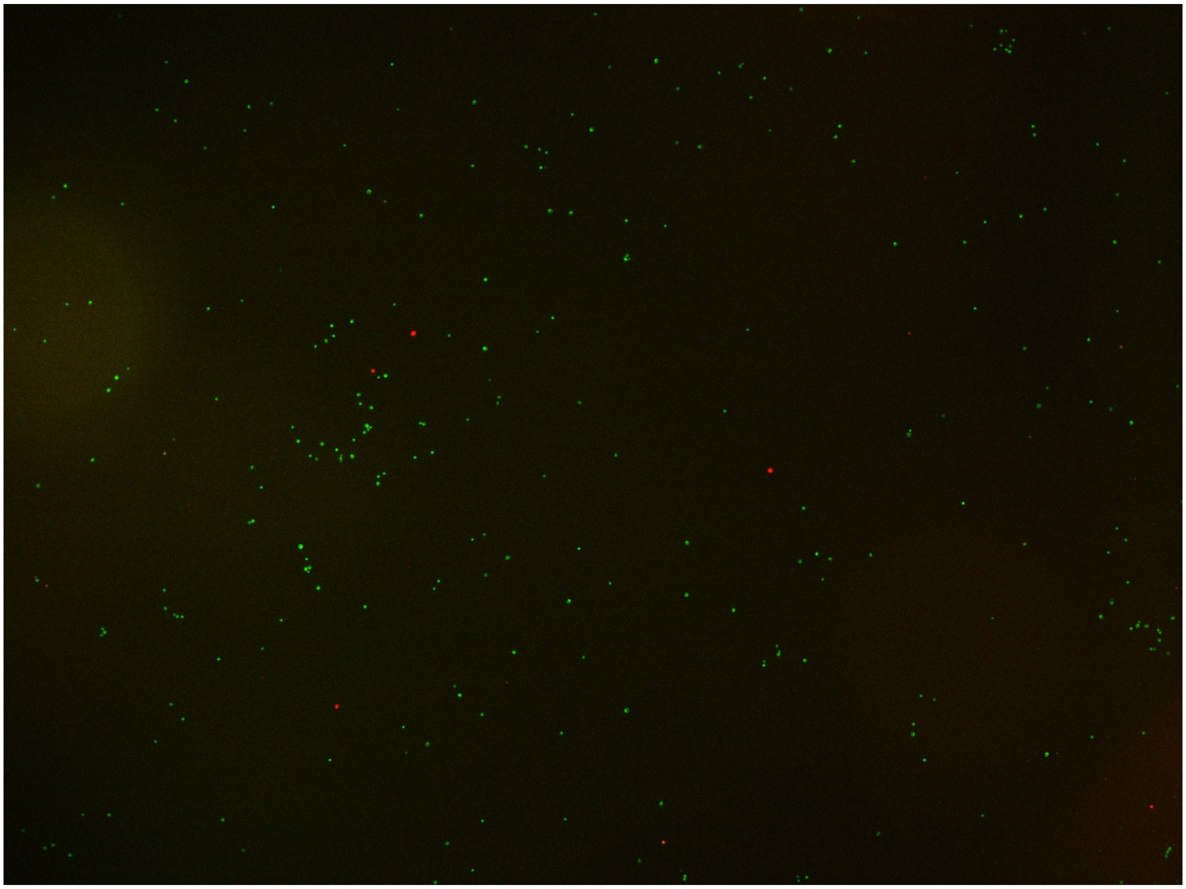

Figure S 13: Combination of the green (all cells) and red (dead cells) fluorescence channels before the experiments. Obviously, most cells show only green fluorescence, indicating that prior to the experiments almost all cells are alive. Image taken using a 10x EC Epiplan objective lense (Carl Zeiss) on an upright microscope (Carl Zeiss Axioscope A.1), and red and green fluorescence filter sets. Pictures taken with a Lumenera Infinity 3S-1URM monochrome camera with 10 ms exposure time and combined using ImageJ.

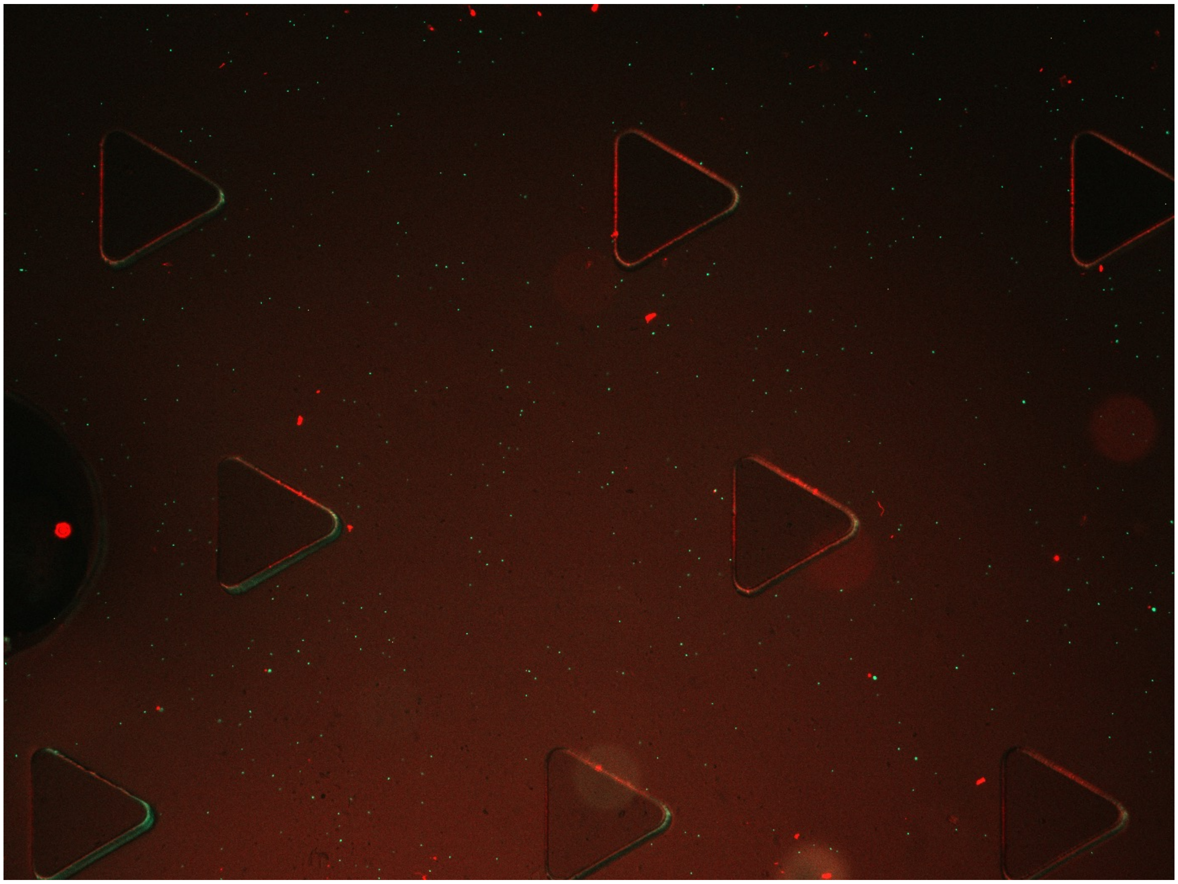

Figure S 14: Combination of the green (all cells) and red (dead cells) fluorescence channels of the released cells of an experiment performed with 150 V_{RMS} and at a flow rate of 1 mL/min. The cell suspension was filled in a PDMS microchannels as used for the microchannel experiments in the paper. The triangular support structures can be seen in the picture. Obviously, most cells show only green fluorescence, indicating a viability of above 95% after the experiments. Image taken using a 5x EC Epiplan objective lense (Carl Zeiss) on an upright microscope (Carl Zeiss Axioscope A.1), and red and green fluorescence filter sets. Note that the cells appear smaller compared to Fig. S13 because the pictures were taken with a 5x lense instead of a 10x lense. A high amount of background fluorescence is visible since the residual dye was not washed off. Pictures taken with a Lumenera Infinity 3S-1URM monochrome camera with 30 ms exposure time and combined using ImageJ.

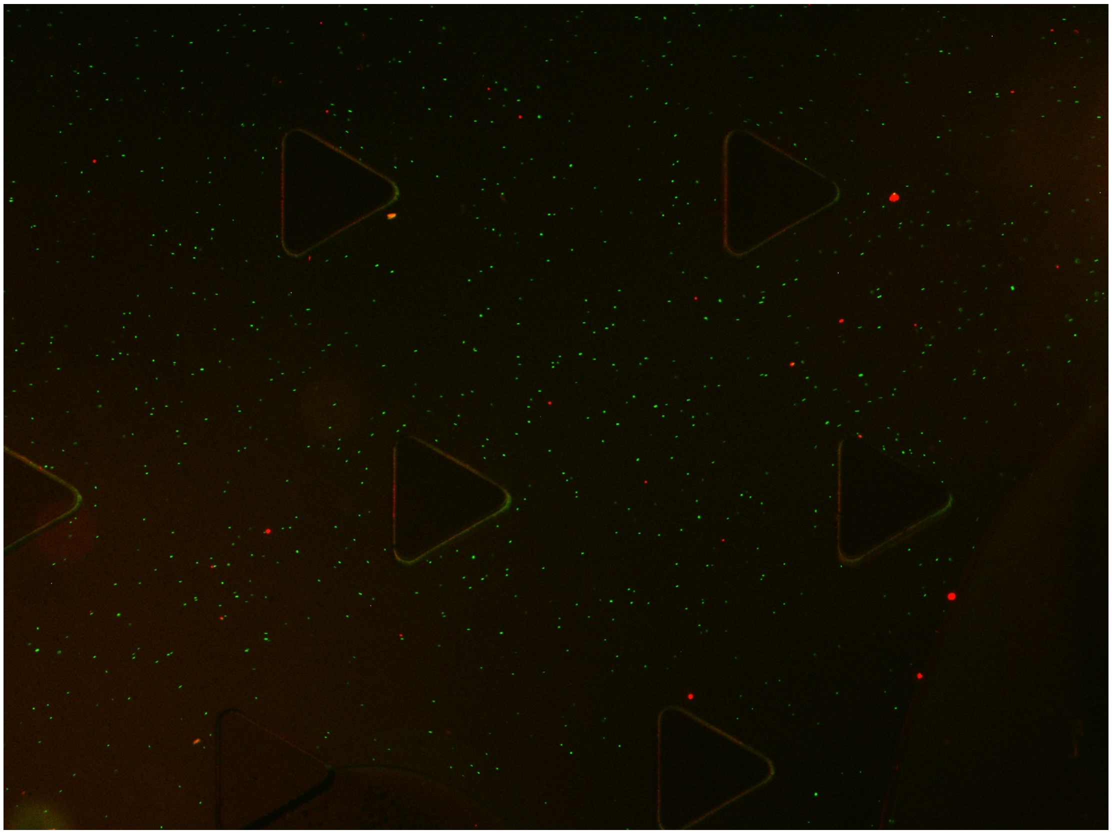

Figure S 15: Combination of the green (all cells) and red (dead cells) fluorescence channels of the released cells of an experiment performed with 300 V_{RMS} and at a flow rate of 6 mL/min. The cell suspension was filled in a PDMS microchannels as used for the microchannel experiments in the paper. The triangular support structures can be seen in the picture. Obviously, most cells show only green fluorescence, indicating a viability of above 95% after the experiments. Image taken using a 5x EC Epiplan objective lense (Carl Zeiss) on an upright microscope (Carl Zeiss Axioscope A.1), and red and green fluorescence filter sets. Note that the cells appear smaller compared to Fig. S13 because the pictures were taken with a 5x lense instead of a 10x lense. A high amount of background fluorescence is visible since the residual dye was not washed off. Pictures taken with a Lumenera Infinity 3S-1URM monochrome camera with 30 ms exposure time and combined using ImageJ.

8. Supplementary Movie S1

The supplementary movie S1 shows trapping of 5*μ*m PS particles (we used 5 *μ*m in the video compared to 1 *μ*m in the experiments for better visualization) at 0.2 mL/h throughput under the application of 1050 V_{RMS} in a microchannel with h_S = 262 *μ*m and d = 100 *μ*m. Firstly, the electric field is off and particles travel through the channel unaffected. After application of the field, all particles are quickly trapped at field maxima around the post. After the field is turned off, particles are released.
